# *Betacoronavirus* Genomes: How Genomic Information has been Used to Deal with Past Outbreaks and the COVID-19 Pandemic

**DOI:** 10.3390/ijms21124546

**Published:** 2020-06-26

**Authors:** Alejandro Llanes, Carlos M. Restrepo, Zuleima Caballero, Sreekumari Rajeev, Melissa A. Kennedy, Ricardo Lleonart

**Affiliations:** 1 Centro de Biología Celular y Molecular de Enfermedades, Instituto de Investigaciones Científicas y Servicios de Alta Tecnología (INDICASAT AIP), Panama City 0801, Panama; allanes@indicasat.org.pa (A.L.); crestrepo@indicasat.org.pa (C.M.R.); zcaballero@indicasat.org.pa (Z.C.); 2 College of Veterinary Medicine, University of Florida, Gainesville, FL 32610, USA; sree63rajeev@gmail.com; 3 College of Veterinary Medicine, University of Tennessee, Knoxville, TN 37996, USA; mkenned2@utk.edu

**Keywords:** betacoronaviruses, genomics, SARS-CoV, MERS-CoV, SARS-CoV-2, COVID-19

## Abstract

In the 21st century, three highly pathogenic betacoronaviruses have emerged, with an alarming rate of human morbidity and case fatality. Genomic information has been widely used to understand the pathogenesis, animal origin and mode of transmission of coronaviruses in the aftermath of the 2002–2003 severe acute respiratory syndrome (SARS) and 2012 Middle East respiratory syndrome (MERS) outbreaks. Furthermore, genome sequencing and bioinformatic analysis have had an unprecedented relevance in the battle against the 2019–2020 coronavirus disease 2019 (COVID-19) pandemic, the newest and most devastating outbreak caused by a coronavirus in the history of mankind. Here, we review how genomic information has been used to tackle outbreaks caused by emerging, highly pathogenic, betacoronavirus strains, emphasizing on SARS-CoV, MERS-CoV and SARS-CoV-2. We focus on shared genomic features of the betacoronaviruses and the application of genomic information to phylogenetic analysis, molecular epidemiology and the design of diagnostic systems, potential drugs and vaccine candidates.

## 1. Introduction

Coronaviruses (CoV) are important pathogens of vertebrates with the ability to cause respiratory, enteric and systemic diseases in humans and animals. They are enveloped, single-stranded, positive-sense RNA viruses belonging to subfamily *Orthocoronavirinae* of family *Coronaviridae*, order *Nidovirales*. The subfamily is further divided into four genera, namely, *Alphacoronavirus*, *Betacoronavirus*, *Gammacoronavirus* and *Deltacoronavirus*. The majority of clinically relevant coronaviruses belong to the *Alphacoronavirus* and *Betacoronavirus* genera [1]. Genus *Alphacoronavirus* comprises species infecting a diverse group of mammals, including two (229E and NL63) of the seven known species of human coronaviruses. In the case of genus *Betacoronavirus*, the International Committee on Taxonomy of Viruses (ICTV) currently divides it into five subgenera, *Embecovirus*, *Sarbecovirus*, *Merbecovirus*, *Nobecovirus* and *Hibecovirus*, established based on phylogenetic analysis of conserved protein domains (see Basic phylogenetic relationships). The first four of these subgenera were formerly known as lineages or subgroups A, B, C and D, respectively.

Subgenus *Embecovirus* includes two human coronaviruses (HKU1 and OC43), as well as several animal coronaviruses of veterinary relevance such as bovine, canine, equine, porcine and murine coronaviruses. *Sarbecovirus* comprises the severe acute respiratory syndrome (SARS)-related coronaviruses such as SARS-CoV and SARS-CoV-2 (2019-nCoV), respectively responsible for the 2002–2003 SARS outbreak and the 2019–2020 coronavirus disease 2019 (COVID-19) pandemic. Several SARS-related bat coronaviruses, mainly isolated from Chinese horseshoe bats (*Rhinolophus* spp.), also belong to this subgenus. Subgenus *Merbecovirus* comprises the Middle East respiratory syndrome (MERS)-related coronaviruses, including the MERS-CoV responsible for the 2012 MERS outbreak, as well as two additional species of bat coronaviruses isolated from *Tylonycteris* and *Pipistrellus* bats. Subgenera *Nobecovirus* and *Hibecovirus* comprise only bat coronaviruses, mainly isolated from *Rousettus* and *Hipposideros* bats, respectively.

Since the 2002–2003 SARS outbreak, genomic information has become ever-increasingly significant to address outbreaks caused by pathogenic coronaviruses. Before the 2019–2020 COVID-19 pandemic, there were ~1200 complete genomes of betacoronaviruses deposited in the GenBank database. The number of available genomes has increased dramatically during the pandemic, with more than 6000 complete genomes available in Genbank as of June 2019, and almost 50,000 genomic sequences in other public repositories. A variety of information including phylogenetic relationships, mode of transmission, evolutionary rates and the role of mutations in infection and disease severity can be deduced from comparing multiple genomes. In this review, we focus on the genomic features of family *Coronaviridae* with special emphasis on the *Betacoronavirus* genus. We also review how genomic information can be useful to tackle epidemics caused by these viruses, including the ongoing COVID-19 pandemic and future ones, potentially caused by emerging strains.

## 2. Genome Structure and Protein-Coding Genes

**Figure 1:**
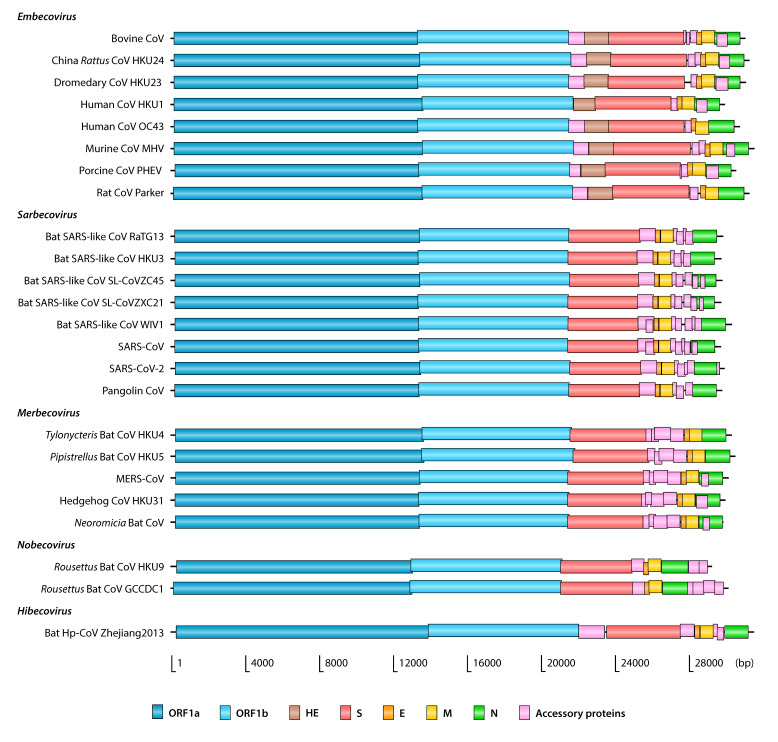
Organization of betacoronavirus genomes. Name abbreviations are provided in Table 1.

Betacoronaviruses, like all other members of the *Coronaviridae* family, have relatively large RNA genomes of around 30 kb in size (Table 1). The genomes have short untranslated regions (UTR) at both ends, with a 5′ methylated cap and a 3′ polyadenylated tail. Typically, genomes contain 9–12 open reading frames (ORF) (Figure 1), six of which are conserved and follow the same order. These conserved ORFs encode the replicase/transcriptase polyproteins and the spike (S), envelope (E), membrane (M) and nucleocapsid (N) structural proteins. Replicase/transcriptase is organized in two overlapping ORFs, called ORF1a (11–13 kb) and ORF1b (7–8 kb), that occupy nearly two thirds of the genome. These ORFs are translated into two polyproteins that later cleave themselves to form several nonstructural proteins (Nsps), most of them involved in genome replication and transcription [2]. The remaining 3′ portion of the genome encodes the structural proteins and the so-called accessory proteins, whose number and functions vary among different coronaviruses.

**Table 1:** Genomic features of representative betacoronaviruses.

Virus ^1^	GenBank Accession	Size (bp)	GC%	ORFs/Accessory Proteins ^2^
*Embecovirus*				
Bovine CoV	NC_003045	31,028	37.12	12/5
China *Rattus* CoV HKU24	NC_026011	31,249	40.07	11/4
Dromedary CoV HKU23	KF906249	31,052	36.95	9/2
Human CoV HKU1	NC_006577	29,926	32.06	9/2
Human CoV OC43	NC_006213	30,741	36.79	9/2
Mouse hepatitis virus (MHV)	NC_001846	31,526	42.03	11/4
Porcine hemagglutinating encephalomyelitis virus (PHEV)	DQ011855	30,480	37.25	12/5
Rat CoV Parker	NC_012936	31,250	41.26	10/3
*Sarbecovirus*				
Bat SARS-like CoV RaTG13	MN996532	29,855	38.04	11/5
Bat SARS-like CoV HKU3	DQ022305	29,728	41.12	12/6
Bat SARS-like CoV SL-CoVZC45	MG772933	29,802	38.90	12/6
Bat SARS-like CoV SL-CoVZXC21	MG772934	29,732	38.82	12/6
Bat SARS-like CoV WIV1	KF367457	30,309	40.77	13/7
SARS-CoV (Human)	NC_004718	29,751	40.76	14/8
SARS-CoV (Civet)	AY686863	29,499	40.85	13/7
SARS-CoV-2 (Human)	NC_045512	29,903	37.97	12/6
SARS-CoV-2 (Tiger)	MT365033	29,897	37.97	11/5
Pangolin CoV	MT040333	29,805	38.52	10/4
*Merbecovirus*				
Hedgehog CoV HKU31	MK907286	29,951	37.69	10/4
MERS-CoV (Human)	NC_019843	30,119	41.24	11/5
MERS-CoV (Dromedary camel)	KF917527	29,851	41.19	10/4
*Neoromicia* bat CoV	MF593268	30,009	40.21	10/4
*Pipistrellus* bat CoV HKU5	NC_009020	30,482	43.19	10/4
*Tylonycteris* bat CoV HKU4	NC_009019	30,286	37.82	10/4
*Nobecovirus*				
*Rousettus* bat CoV GCCDC1	NC_030886	30,161	45.30	11/5
*Rousettus* bat CoV HKU9	NC_009021	29,114	41.05	9/3
*Hibecovirus*				
Bat Hp-BetaCoV Zhejiang2013	NC_025217	31,491	41.28	10/4

^1^ Detailed information about the corresponding isolates is provided in Supplementary Table S1. ^2^ Number of open reading frames (ORFs) annotated in the corresponding GenBank entry. This number can vary among different versions of genome annotation for the same isolate.

**Figure 2:**
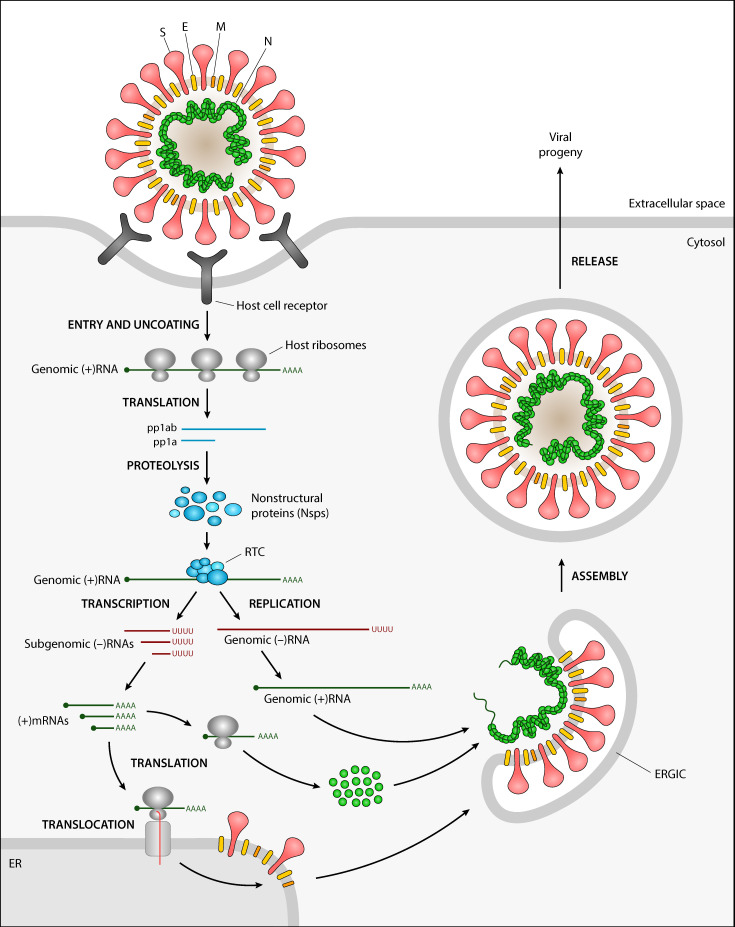
Replication cycle of a typical coronavirus. Upon recognition of the host cell receptor, the viral particle enters the host cell and is uncoated, releasing its positive-sense genomic RNA. Host ribosomes translate polyproteins pp1a and pp1ab, which self-cleave to produce the nonstructural proteins (Nsps). Several Nsps assemble into the replicase-transcriptase complex (RTC) that generates the mRNAs for structural and accessory proteins through transcription, as well as positive-sense genomic RNAs through replication. Viral core particles are assembled within smooth vesicles derived from the endoplasmic reticulum-Golgi intermediate compartment (ERGIC). The viral progeny is ultimately released via exocytosis.

**Figure 3:**
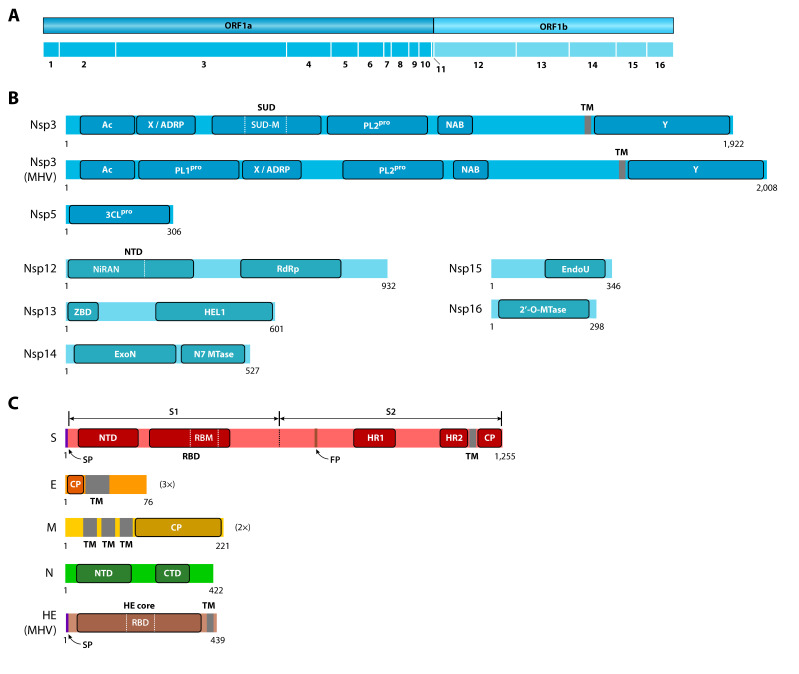
Main functional domains in protein-coding genes. (**A**) Location of Nsps along the sequence of ORF1a and ORF1b. (**B**) Functional domains of Nsp3, Nsp5, Nsp12, Nsp13, Nsp14, Nsp15 and Nsp16. (**C**) Functional domains of structural proteins. All proteins are from SARS-CoV, except for the Nsp3 and HE proteins of murine hepatitis virus (MHV), which are included for comparative purposes. Proteins are drawn to scale, except for E and M, which are drawn three (3×) and two (2×) times larger, respectively. Specific domain name abbreviations are explained in the main text. TM: transmembrane domain, SP: signal peptide, FP: fusion peptide, RBD: receptor-binding domain, CP: cytoplasmic domain, NTD: N-terminal domain, CTD: C-terminal domain.

As with most viruses, coronavirus genomes are compact and only encode the proteins required for the viral replication cycle (Figure 2). Transcription of the protein-coding genes involves the production of subgenomic mRNAs containing a common leader sequence in their 5′ end. This common leader is in turn encoded near the 5′ end of the genome and its fusion to subgenomic mRNAs is mediated by a conserved transcription regulatory sequence (TRS) preceding most genes [2]. The role of viral proteins in the replication cycle and their conserved domains (Figure 3) are briefly reviewed in the sections below. We have also provided the InterPro accession numbers for these domains, if available.

### 2.1. Spike (S) Protein

Spike (S) is a glycoprotein that recognizes the host cell receptor and allows the virus to attach to the surface of host cells. Its name refers to the spike-like structures located in the outer surface of the viral envelope, which are trimers of the S protein. After receptor recognition and attachment, the virus enters the host cell through endocytosis or by direct fusion of its envelope with the host cell plasma membrane. SARS-CoV and SARS-CoV-2 use angiotensin-converting enzyme 2 (ACE2) as their receptor [3,4], whereas MERS-CoV uses dipeptidyl peptidase 4 (DPP4) [5] and murine coronaviruses use the murine carcinoembryonic antigen-related adhesion molecule 1 (mCEACAM1a) [6,7]. Viruses from the *Embecovirus* subgenus can use certain types of sialic acids as receptors [8], due to an additional hemagglutinin esterase gene uniquely present in this subgenus (discussed below).

Due to its binding specificity, the S protein determines tissue tropism and host species range of different coronaviruses. Binding specificity of the S protein is determined by its receptor-binding domain (RBD) (IPR018548), sometimes called C-domain, responsible for recognizing and binding to the host cell receptor. The S protein sequence is commonly divided into two sections, termed S1 and S2, corresponding to the two subunits in which the protein is cleaved by host proteases after receptor recognition, although this cleavage does not occur in all coronaviruses [9]. The RBD is located in the S1 subunit and contains a shorter receptor-binding motif (RBM) that directly interacts with the receptor. Consistent with the fact that SARS-CoV and MERS-CoV have different host cell receptors, their RBDs are structurally similar, but the RBMs differ in sequence [10]. The RBMs of SARS-CoV and SARS-CoV-2 are composed of ~70 amino acids [11,12], whereas the MERS-CoV RBM is composed of ~83 amino acids [13,14]. The S1 subunit also contains an additional N-terminal domain (NTD) (IPR032500) that has been shown to mediate binding to mCEACAM1a in murine coronaviruses [15].

The S2 subunit is considered to act as a class I viral fusion protein, promoting virus entry to the host cell through membrane fusion [16]. This subunit contains a fusion peptide (FP) that is believed to penetrate the host cell membrane, initiating the membrane fusion process [17]. S2 contains two additional α-helical heptad repeat domains, called HR1 and HR2 (IPR027400), which interact with each other to form a coiled coil conformation, facilitating membrane fusion by bringing together the viral envelope and the host cell membrane [18]. The S2 subunit also contains a transmembrane (TM) domain that anchors the S protein to the viral envelope, as well as a short cysteine-rich endodomain, also known as CP (cytoplasmic) domain, oriented towards the interior of the viral particle.

### 2.2. Replicase/Transcriptase and Nonstructural Proteins

Upon host cell entry, the virus is uncoated and the host ribosome then translates the first two overlapping ORFs, ORF1a and ORF1b, to generate the replicase/transcriptase polyproteins pp1a and pp1ab. The pp1a polyprotein is synthesized by translation of ORF1a, whereas the longer pp1ab polyprotein is synthesized from both ORFs, due to a ribosomal frameshifting event allowing their continuous translation [2]. These polyproteins self-cleave to produce up to 16 nonstructural proteins (Nsps) (see Snijder et al. [19] for a comprehensive review). Nsp1 to Nsp11 are encoded by ORF1a and are therefore present in both pp1a and pp1ab, whereas Nsp12 to Nsp16 are encoded by ORF1b and are only present in pp1ab.

At least two Nsps are responsible for the proteolytic activity, namely, Nsp3 (papain-like protease or PL^pro^) and Nsp5 (3C-like protease or 3CL^pro^). The Nsp3 proteins from genus *Alphacoronavirus* and subgenus *Embecovirus* have two PL^pro^ functional domains (IPR022733), respectively termed PL1^pro^ and PL2^pro^. All other coronaviruses have only one domain, collinear with PL2^pro^. Nsp3 from SARS-related coronaviruses has several functional domains in addition to PL2^pro^, including an acidic (Ac) C-terminal domain, an ADP-ribose-1’’-phosphatase (ADRP) domain, a SARS-specific unique domain (SUD) (IPR024375), a nucleic acid-binding (NAB) domain (IPR032592) and a TM segment [20]. At least two of these domains seem to have affinity for single-stranded RNA [21,22].

Several Nsps (Nsp7 to Nsp16) form the active multimeric replicase/transcriptase complex (RTC). The main component of this complex is Nsp12, the RNA-dependent RNA polymerase (RdRp) that directly mediates the de novo primer-independent RNA synthesis during replication of the virus, as well as transcription of ORFs to produce the mRNAs for structural and accessory proteins. During replication, RdRp synthesizes a negative-sense genomic RNA by using the positive-sense genome as a template. During transcription, RdRp synthesizes negative-sense subgenomic RNAs that are subsequently transcribed into the corresponding positive-sense mRNAs. These mRNAs are then translated by the host ribosome into the structural and accessory proteins. To accomplish these functions in transcription and replication, Nsp12 has at least two well-conserved functional domains, namely, the RdRp catalytic domain (IPR007094) and a relatively large N-terminal domain (NTD) (IPR009469). This NTD is unique to the *Nidovirales* order and contains a nucleotidyltransferase subdomain called NiRAN (nidovirus RdRp-associated nucleotidyltransferase) [23].

The other Nsps forming the RTC assist RdRp during replication and transcription [24]. Nsp7 and Nsp8 are thought to help with the processivity of RdRp and together form the main polymerase holoenzyme [25]. Nsp13 is a highly conserved helicase subunit that is required for efficient replication of the viral genome [19]. In addition to the HEL1 helicase core domain (IPR027351), Nsp13 also has an N-terminal cysteine-rich zinc-binding domain (ZBD) (IPR027352) that appears to modulate the helicase activity [26]. Three additional nonstructural proteins, Nsp14, Nsp15 and Nsp16, have functional domains likely to be involved in RNA processing pathways. Nsp14 is a bifunctional protein that has a N7-methyltransferase domain and an ExoN domain with 3′-5′ exonuclease activity. This exonuclease activity provides a proofreading function that is lacking in RdRp and enhances the fidelity of replication [27].

### 2.3. Envelope (E) and Membrane (M) Proteins

Envelope (E) and membrane (M) are conserved, envelope-associated, integral membrane proteins. Proteins S, E and M are translocated into the endoplasmic reticulum (ER) of the host cell during translation. Unlike the S protein, however, E and M do not appear to have a recognizable N-terminal signal peptide [9]. Upon entry into the ER, the three proteins are integrated into the ER membrane and follow the secretory pathway towards the ER-Golgi intermediate compartment (ERGIC). There, E and M engage in several molecular interactions to facilitate assembly and release of new viral particles [28,29,30].

The E protein has a single TM domain and a relatively short N-terminal CP endodomain, whereas M has three TM domains and a much larger C-terminal CP endodomain. It has been suggested that both CP endodomains play a significant role in the critical functions of these proteins in assembly and release of new viral particles [28,31]. The E protein also acts as an ion channel, an activity that has been associated with its TM domain [32,33]. In SARS-CoV, this activity is not essential for replication, but it appears to be required for virulence [34].

### 2.4. Nucleocapsid (N) Protein

The nucleocapsid (N) protein binds to genomic RNAs in a beads-on-a-string conformation. Unlike S, E and M, the N protein stays in the cytosol of the host cell after translation, where it binds genomic RNAs to form new nucleocapsids. These nucleocapsids travel to the ERGIC and are used for the assembly of new viral core particles. The N protein also appears to bind to Nsp3 and M, thus suggesting an important role in guiding viral RNA through replication, transcription and assembly [9]. The N protein contains two functional domains, termed N-terminal domain (NTD) (IPR037195) and C-terminal domain (CTD) (IPR037179), both of which are capable to interact with RNA [35,36].

### 2.5. Accessory Proteins

Accessory proteins are genus- or species-specific and are usually dispensable for viral replication in vitro but required in vivo [37]. The functions of accessory proteins and their pathophysiological roles are not completely understood. SARS-CoV contains at least eight ORFs encoding accessory proteins, namely, ORFs 3a, 3b, 6, 7a, 8a, 8b and 9b. Some of these proteins, particularly 6 and 7b, appear to contribute to virulence [38,39]. Most of these proteins are involved in cellular processes such as interfering with DNA synthesis, induction of caspase-dependent apoptosis, induction of proinflammatory cytokines and activation of the mitogen-activated protein kinase (MAPK) pathway [37]. Many of these accessory proteins are also incorporated into mature SARS-CoV virions, filling the role of minor structural proteins. The SARS-CoV-2 genome seems to encode a set of accessory proteins similar to that of SARS-CoV, with noticeable differences in ORFs 3a, 3b and 8b, which have been associated with interferon modulation and activation of the inflammasome [40].

Certain coronaviruses have one or two ORFs overlapping the N protein gene, although these are not always annotated in the corresponding genomes. Betacoronaviruses from the *Embecovirus* and *Merbecovirus* subgenera typically have a single overlapping ORF that has been found to encode a 23-kDa protein in mouse hepatitis virus (MHV) [41] and bovine coronavirus [42]. Experiments performed in MHV-infected cells have demonstrated that this protein is a structural component of the MHV virion and may be involved in the processing or transport of the S protein [41]. In the *Sarbecovirus* subgenus, there are usually one or two shorter ORFs overlapping the N gene, often termed ORF9b and ORF9c. ORF9b has been shown to encode an accessory protein that is also a virion component [43] and seems to participate in the suppression of host innate immunity [44].

### 2.6. Haemagglutinin Esterase (HE)

All known betacoronaviruses from the *Embecovirus* subgenus have an additional haemagglutinin esterase (HE) gene located upstream of that encoding the S protein [45]. This gene encodes a glycoprotein with neuraminate O-acetylesterase activity that mediates reversible attachment to O-acetylated sialic acids by acting as a receptor-binding molecule and a receptor-destroying enzyme [46]. HE is an integral membrane protein with a single TM domain and a relatively large ectodomain, which contains the esterase core domain (IPR003860) and a lectin subdomain acting as the RBD [47]. This gene is suspected to be acquired from the influenza C virus through heterologous recombination [48]. Due to the absence of this gene in all other betacoronaviruses, this event is likely to have occurred after major subgenera diverged from common ancestors.

## 3. Basic Phylogenetic Relationships

Phylogenetic analyses help us understand the evolutionary history of viruses and provide a solid basis for their classification. A paramount application of these analyses is to make inferences about the origin of novel viral strains or species. This is particularly relevant in the context of outbreaks, to identify possible animal reservoirs involved in transmission to other susceptible hosts, including humans. To perform these analyses, researchers usually focus on genes or genomic segments conserved through all the species of interest, but with enough sequence divergence to allow their unambiguous separation in a phylogenetic tree. In the case of the coronaviruses, phylogenetic analyses are usually based on whole or partial sequences of the ORF1ab, S and N genes, whereas genes E and M are generally deemed as too short for these analyses [45].

**Figure 4:**
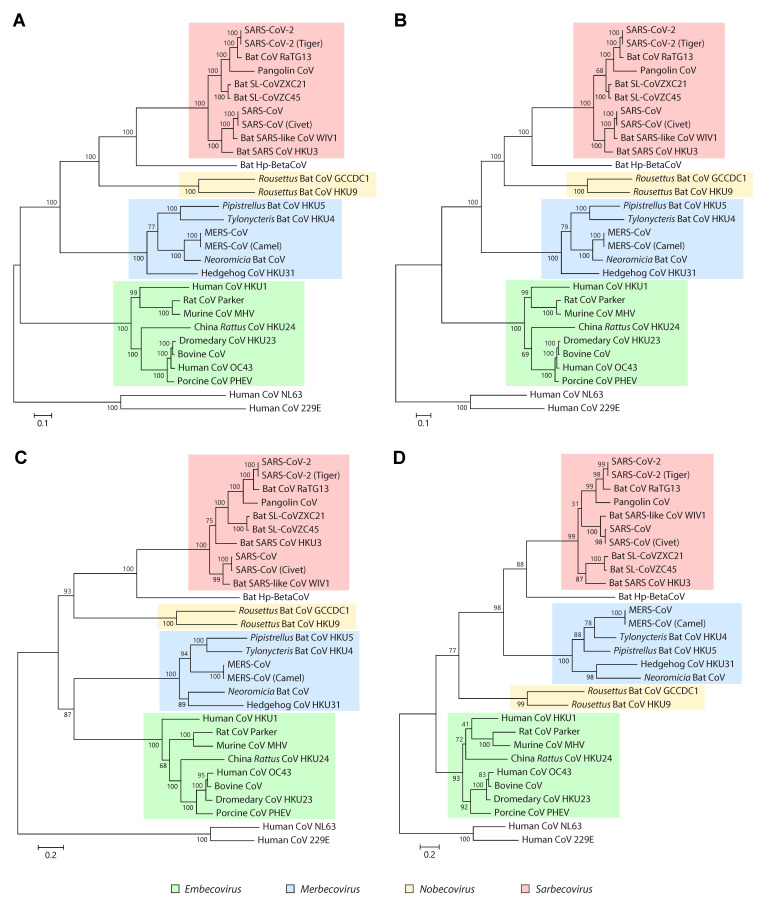
Phylogenetic analysis of representative betacoronaviruses. Figure shows four alternative phylogenies for coronaviruses in Table 1, inferred from complete genome sequences (**A**), concatenated sequences of ORF1ab domains (**B**), whole S protein (**C**) and the receptor-binding domain (RBD) (**D**). Phylogenetic analysis was performed as previously described [4,52], briefly, sequences were aligned with MAFFT [56] and trees were built with IQ-TREE [57], with the maximum likelihood (ML) method and the GTR+G+I model. For protein sequences, amino acid alignments were converted to nucleotides with PAL2NAL [58]. Numbers above or below branches indicate branch support measures expressed as percentage and estimated using the Shimodaira-Hasegawa (SH)-like approximate likelihood ratio test (aLRT) with 1000 replicates. Trees were rooted with human alphacoronaviruses 229E and NL63 (GenBank accession numbers NC_002645 and NC_005831, respectively).

Several genes are usually considered when exploring the phylogenetic relationships among coronaviruses, since trees built from different genomic regions often have inconsistent topologies [45] (Figure 4). One possible cause for these inconsistencies is genetic recombination, which is thought to occur frequently during evolution of coronaviruses (see Molecular epidemiology). Recombination usually involves segments totally or partially spanning the S gene but may be associated with other regions of the genome. For instance, soon after the discovery of human coronavirus HKU1 in 2005, phylogenetic analysis suggested the existence of two putative genotypes, but conflicting results were obtained when using different regions of the genome to infer the phylogenetic relationship between these genotypes [49,50]. It was later demonstrated that these discrepancies were due to a recombination between the two genotypes, with recombination breakpoints located within Nsp16 and HE [51].

For the taxonomic classification of coronaviruses, the ICTV currently recommends the use of domains 3CL^pro^, NiRAN, RdRp, ZBD and HEL1 of Nsp3, Nsp12 and Nsp13 [52]. These domains are conserved in all viruses of the order *Nidovirales* and can therefore be used for deeper phylogenetic analyses [53]. Recent studies exploring the phylogenetic position of SARS-CoV-2 have shown that trees built using some of these conserved domains are consistent with those based on whole genome sequences, at least at the genus and subgenus levels [4,52,54,55] (Figure 4A,B). However, in studies exploring shallower phylogenetic relationships, such as those focused on closely related viral strains isolated from different hosts, conserved domains may not have enough variability to ensure robust separation of some taxa. This is evidenced by the relatively low branch support estimates occasionally obtained for some subclades from the same subgenus in trees based on conserved domains, when compared to whole genome trees [4,52] (Figure 4B,D). More variable segments or complete genome sequences may be a better choice in these scenarios, to build more robust phylogenetic trees.

Several studies addressing the origin of coronavirus species have identified bats as the natural reservoirs of alphacoronaviruses and betacoronaviruses [59,60]. This is not surprising, since bat coronaviruses are highly ubiquitous in the most currently accepted taxonomic subgroups [1]. In fact, five of the seven known human coronaviruses are likely to have originated from bats, namely, NL63, 229E, MERS-CoV, SARS-CoV and SARS-CoV-2. The remaining two human coronaviruses, HKU1 and OC43, are thought to have originated from rodents [61]. In the case of SARS-CoV, its possible origin from bats was first suggested in 2005, when two studies independently reported the discovery of SARS-related coronaviruses isolated from Chinese horseshoe bats (*Rhinolophus* spp.) [62,63], with several more strains discovered in subsequent years (reviewed by Luk et al. [64]). Similar findings have been reported for MERS-CoV, which was found to be closely related to coronaviruses isolated from bamboo bats (*Tylonycteris* spp.) and pipistrelle bats (*Pipistrellus* spp.), respectively termed *Tylonycteris* bat coronavirus HKU4 (Ty-BatCoV-HKU4) and *Pipistrellus* bat coronavirus HKU5 (Pi-BatCoV-HKU5) [65]. In phylogenetic trees, these two coronaviruses separate well from MERS-related coronaviruses found in other bat species, including those recently isolated from serotine bats (*Neoromicia* spp.) in South Africa [66].

Another important conclusion drawn from exhaustive phylogenetic analyses is that, although bats appear to act as natural reservoirs of coronaviruses, intermediate animal hosts may also play a critical role in transmission to other susceptible hosts. The presumed intermediate host for SARS-CoV, the masked palm civet from the *Viverridae* family (*Paguma larvata*), was identified even before the natural bat carriers, when highly similar SARS-CoV strains were found in the civets from a wet market and in workers supposed to handle them [67]. The intermediate role of civets was suspected when comparing samples from market civets to those in the wild, which suggested that SARS-CoV was likely transmitted to the market civets by other animals [68]. A similar scenario has been reported for MERS-CoV and its intermediate host, the dromedary camel (*Camelus dromedarius*). After the 2012 MERS outbreak, MERS-CoV strains highly similar in sequence to those from human patients were isolated from camels [69,70]. In 2013, a novel dromedary camel coronavirus HKU13 was also identified [71]; however, phylogenetic analysis positioned it within the *Embecovirus* subgenus, and it is therefore not directly related to MERS-CoV.

Soon after the onset of the 2019–2020 COVID-19 pandemic and the availability of the whole genome sequence of the novel coronavirus, phylogenetic analysis revealed that it was closely related to SARS-CoV and it was officially designated as SARS-CoV-2 [52]. Not surprisingly, it was soon reported that SARS-CoV-2 was phylogenetically related to two bat SARS-like coronaviruses, SL-CoVZC45 and SL-CoVZXC21, previously isolated from *Rhinolophus sinicus* in 2018 [54,72,73]. Bat coronavirus RaTG13, isolated from *Rhinolophus affinis* in 2013, was found to be even more closely related to SARS-CoV-2 than the first two [4]. Further studies identified coronaviruses similar to SARS-CoV-2 in Malayan pangolins (*Manis javanica*), a highly smuggled animal illegally sold in China [55,74]. Although bat coronavirus RaTG13 is phylogenetically closer to SARS-CoV-2 than the pangolin coronaviruses, the RBDs of the latter are more similar to those of SARS-CoV-2, thus suggesting a possible role of pangolins as intermediate hosts in transmission to humans [75].

## 4. Molecular Epidemiology

Molecular epidemiology focuses on the contribution of genomic, genetic and other molecular factors to etiology, distribution and prevention of diseases. Central to molecular epidemiology of betacoronaviruses is their circulation among different animal hosts, as well as the evolutionary forces that facilitate these cross-species jumps. Here, we discuss how genomic information has been used to better understand the rate of evolution of betacoronaviruses and their transmission in human populations, as well as the evolutionary changes associated with host and tissue tropism.

### 4.1. Evolutionary Rates and Divergence

Estimation of evolutionary rates is an important step to characterize the genetic diversity among viral lineages and to place a timescale in phylogenetic hypotheses explaining their origin and divergence. The rate of evolution of viruses is often assessed through the number of errors occurring during replication of the viral genome (the mutation rate) and the frequency at which such mutations become fixed in the population (the substitution rate) [76]. The substitution rate depends on several factors, including the underlying mutation rate and the presence of selective forces that influence fixation of mutations in association with their fitness. Mutation rates of RNA viruses are generally higher than those of DNA viruses, due to the lack of a proofreading activity and consequent low fidelity of their RdRp [77]. However, due to the proofreading activity of Nsp14, members of the order *Nidovirales* have relatively lower mutation rates [78].

The substitution rate is often expressed in substitutions per nucleotide site per year (s/n/y) and can be estimated from phylogenetic reconstructions when divergence time is known for particular lineages. Although several methods have been traditionally used to estimate substitution rates, including linear regression and maximum likelihood (ML), the most popular method nowadays is the Bayesian Markov chain Monte Carlo (MCMC) approach, such as that implemented in the BEAST package [79]. Globally, substitution rates of coronaviruses have been estimated to be in the order of 10^−3^–10^−4^ s/n/y [80,81]. Studies conducted in SARS-CoV and MERS-CoV have estimated whole genome substitution rates to be between 0.80–2.38 × 10^−3^ and 0.88–1.37 × 10^−3^ s/n/y, respectively [82,83]. However, variation in the estimates for particular genes have been observed for both SARS-CoV [84,85,86,87,88] and SARS-CoV-2 [89,90], suggesting that the S gene and the region between ORF7b and ORF8 may be subjected to positive selective pressure in some lineages. In contrast, more conserved regions of the genome such as ORF1a and ORF1b appear to be under strong negative or purifying selection.

An important application of this type of analysis is the estimation of the time to most recent common ancestor (TMRCA) between two lineages, as an approximate measure of their time since divergence. Several studies have estimated that the SARS-CoV lineage within the SARS-related coronaviruses most probably emerged between 1961–1985, while the civet SARS-CoV strains may have originated around 1986–1995 [86,87,88]. TMRCA estimates for the SARS-CoV and MERS-CoV strains involved in previous outbreaks have been roughly consistent with the dates when the first cases were reported [83,88]. A preliminary study has estimated that the group containing SARS-CoV-2 and its closest bat coronavirus, RaTG13, may have diverged between 40–70 years ago [91].

### 4.2. Recombination, RBD Mutations and Host/Tissue Tropism

Recombination events are often inferred by comparing phylogenetic trees built from different genes or genomic regions, since occurrence of recombination often leads to inconsistent topologies in such trees (see Basic phylogenetic relationships). One of the most popular methods for detecting recombination in viral genomes is bootscan analysis [92]. To use this method, sequences of target genomes are aligned against reference sequences thought to be involved in the recombination events. The alignments are divided into short sequential segments and phylogenetic trees are then built from these segments. Recombination is suspected in segments for which the trees exhibit an alternative topology, involving different reference sequences. Bootscan and other complementary methods, such as sequence similarity plots for the putative recombinant regions, are implemented in packages such as SimPlot [93] or the Recombination Detection Program (RDP) [94]. Studies relying on these methods have documented the occurrence of recombination in several coronavirus genera and have also provided ample evidence supporting the important role of this process in coronavirus cross-species transmission [1,95,96].

The first reported example of natural recombination in human coronaviruses was that occurring between two different HKU1 genotypes [51]. Putative recombination events between genotypes have also been documented for human coronaviruses NL63 [97] and OC43 [98]. In the case of SARS-related and MERS-related coronaviruses, recombination appears to occur among strains infecting several animal hosts, including bats, intermediary hosts and humans (reviewed by Hu et al. [60] and Su et al. [96]). Studies considering several bat SARS-related coronaviruses have suggested the occurrence of recombination in lineages leading to human and/or civet strains of SARS-CoV, with breakpoints often located close or within the S and ORF8 genes [87,99,100]. These findings suggest that recombination between existing strains can result in new strains or species, with possible differences in host and tissue tropism. For instance, bat coronavirus strains Rs3367 (WIV1) and WIV16 have been reported to have high sequence similarity to human/civet SARS-CoV at the S gene, allowing them to use ACE2 as a receptor for cell entry [101,102]. Recombination analysis suggested that at least one civet SARS-CoV strain (SZ3) may have originated by recombination between WIV16 and another bat SARS-CoV strain (Rf4092) [103].

In the case of SARS-CoV-2, comparison of its genome to those of other SARS-related coronaviruses did not provide enough evidence supporting recent recombination as a possible explanation for its origin [4,54,73]. However, two putative breakpoints, possibly derived from a past recombination event, were identified within the S gene and flanking its RBD [73]. Globally, the SARS-CoV-2 genome is more similar to those of bat coronaviruses SL-CoVZXC21 and SL-CoVZC45 however, the region between the two breakpoints was found to be more similar to human/civet SARS-CoV and WIV1. Putative recombination signals have also been reported between SARS-CoV-2, RaTG13 and the pangolin-related coronaviruses [55,74]. Although SARS-CoV-2 is more similar to RaTG13 than to the pangolin coronaviruses, some of the latter have higher sequence similarity to SARS-CoV-2 in the RBD. It has also been suggested that these similarities at the amino acid level may be due to convergent evolution, arising from positive selection instead of recombination [74,104].

The fact that different evolutionary events often involve the RBD is likely to be associated with the role of this domain and its RBM in receptor recognition and adaptation to different animal hosts. In SARS-CoV-like viruses, six RBD amino acids have been found to be essential for binding to ACE2, five of which differ between SARS-CoV and SARS-CoV-2 [12,105]. Particular sets of RBD mutations appear to be associated with a specific host range for each coronavirus species, as is the case of humans and civets in SARS-CoV or humans and camels in MERS-CoV. Although it has been suggested that SARS-CoV-2 is optimized for binding to human ACE2, it may also infect other animals with highly similar ACE2 homologs such as pigs, ferrets, cats and primates [75,105]. In fact, the S gene of a SARS-CoV-2 strain recently isolated from a tiger (GenBank accession number MT365033) is identical to those of human isolates, both clustering into the same branch in phylogenetic trees (Figure 4).

Comparative sequence analysis has suggested that positive selection may have a role in shaping the evolution of the S protein and the RBD of SARS-CoV and MERS-CoV [84,106,107,108]. The role of natural selection in the evolution of particular genes is typically inferred by computing the ratio of the rates of nonsynonymous to synonymous changes (K_a_/K_s_) between groups or lineages, with a value greater than 1 indicating an overall positive selective pressure. Although evolution of the SARS-CoV genome during the 2002–2003 SARS outbreak was found to be largely neutral or nearly neutral, at least six mutations occurred in the S protein during the early, middle and late phases of the outbreak, all of which were present in the epidemic strain (Urbani) [106,109]. However, the average K_a_/K_s_ values for the early phase were found to be significantly higher than those for the middle and late phases, suggestive of initial positive selection in the S gene, followed by purifying selection and stabilization. Likewise, a recent study has suggested limited episodes of positive selection during divergence of SARS-CoV-2 from RaTG13, although there is still insufficient evidence to associate these changes with its adaptation to humans [110].

### 4.3. Genetic Variation and Transmission in Human Populations

As pathogenic viruses replicate and spread during outbreaks, their genomes accumulate random mutations that can be used to track the spread of the disease, reconstruct their transmission routes and detect lineages with different levels of virulence and transmissibility. There are several methods for tracking mutations and inferring the mode of transmission from genomic data, most of which require the alignment of sequences from new isolates to reference genomes and the subsequent identification of genetic variants such as single nucleotide variants (SNV) and insertion/deletions (indels). The simplest and fastest methods are based on pairwise distances among samples computed from these variants. However, these methods do not consider evolutionary models and can be highly inaccurate when there is substantial divergence between donor and recipients in transmission chains [111]. More advanced methods are based on ML or MCMC approaches, often within a Bayesian framework, applying an explicit model of evolution to phylogenetic estimation. When these methods are combined with sampling dates, estimations of the presence of significant molecular evolution over a sampling period is possible [111]. Such analyses are implemented in packages like TransPhylo [112,113], Phyloscanner [114], Outbreaker2 [115] or Phybreak [113].

The main output of these methods is a transmission tree indicating which individuals infected others. Although a transmission tree cannot be directly inferred from a phylogenetic tree, it must be consistent with the underlying phylogeny. Together, phylogenetic and transmission trees help us trace back the origin of an outbreak, detect multiple introductions of a pathogen into a given territory, identify mutations that define specific lineages and predict the potential existence of unsampled individuals that may have acted as missing transmission links. Due to their relatively recent development, transmission trees based on genomic data were not widely used to study the transmission routes of previous SARS and MERS outbreaks; however, a recent study analyzing data from the 2003 SARS outbreak has provided new insights into its early stages [116].

Progress in genome sequencing technologies has resulted in an exceptionally high number of SARS-CoV-2 genomes sequenced during the 2019–2020 COVID-19 pandemic. In fact, the COVID-19 pandemic is the second one in history, after the 2009 H1N1 influenza pandemic [117,118], for which genomic data have been generated almost in a real-time fashion, allowing a detailed reconstruction of transmission trees. Genome sequences have been made publicly accessible through several repositories, including a data sharing service hosted by the Global Initiative on Sharing All Influenza Data (GISAID) (https://www.gisaid.org/) [119,120]. In addition to public genome repositories, open-source platforms for real-time data visualization and analysis of genomic data are also available, including NextStrain (https://nextstrain.org) and CoV-GLUE (http://cov-glue.cvr.gla.ac.uk). NextStrain is fed with sequences from the GISAID repository and uses the Augur bioinformatics toolkit (https://github.com/nextstrain/augur) for tracking molecular evolution and the Auspice software (https://nextstrain.github.io/auspice/) for interactive visualization of phylogenomic data. Conversely, CoV-GLUE is a web application based on an integrated software environment called GLUE (Genes Linked by Underlying Evolution), designed to create bioinformatic resources based on viral genome sequences [121]. CoV-GLUE is also based on GISAID data and contains a database of replacements and indels that have been found in previously sampled SARS-CoV-2 sequences. New SARS-CoV-2 genome sequences can be loaded into the platform to identify novel or known mutations, assign them to potential lineages and visualize them in a phylogenetic context.

Several attempts have been made to classify circulating strains of SARS-CoV-2 into lineages or genotypes with potential differences in transmissibility and disease severity. Among these, a study comparing 103 SARS-CoV-2 genomes suggested the existence of two lineages, termed L and S, with potential differences in prevalence [104]. Another recent study compared 160 genomes from different countries and suggested the existence of three subtypes, A, B and C, with differences in geographic distribution and prevalence [122]. However, such studies have been criticized for possible sampling biases and misinterpretation of results [123,124,125,126], stressing that caution should be taken when drawing conclusions from genomic analyses. Limited or inappropriate sampling can bias the inference of transmission networks, potentially hiding introduction events and intermediate states and resulting in inaccurate mutation rate estimates [111]. When describing new lineages based on SNVs and other genetic variants, fixation of the corresponding mutations should be first demonstrated in local populations.

## 5. Diagnostics, Drug Design and Vaccine Candidates

Early diagnosis and rapid development of drugs and vaccines targeting emerging viruses are essential to limit their spread, but traditional development approaches are time-consuming and often inefficient. Conversely, sequence-based approaches allow rapid understanding of viral protein function and pathogenesis, as well as the identification of virus-specific factors and targets suitable for drug and vaccine design. Here, we briefly review how genomic information has fueled the development of diagnostic systems and the design of new drug and vaccine candidates. We begin with a brief introduction to the application of genomics in the development of reverse genetic systems, which are key to the previously mentioned fields.

### 5.1. Reverse Genetic Systems

Genomic information has been widely used in the development of reverse genetic systems, allowing the construction of synthetic viral infectious particles and the manipulation of the genetic composition of viruses for research purposes [127]. These systems have proven indispensable for the characterization of human and animal betacoronaviruses, especially when there is limited access to clinical isolates or for research institutions that do not have the appropriate containment facilities [128]. Reverse genetics was initially difficult to implement for betacoronaviruses due to the relatively large size of their viral RNA genome, which affects transfection efficiency and stability in standard bacterial vectors [127]. Although several systems can be used to construct synthetic coronavirus genomes (reviewed by Almazán et al. [129]), most studies focusing on betacoronaviruses have used a sub-cloning strategy based on in vitro ligation, originally developed for the transmissible gastroenteritis virus (TGEV) [130]. This system is based on the systematic and precise assembly of complete cDNA genomes from a panel of cDNA cassettes that span the entire viral genome and that are flanked by native or engineered specific restrictions sites, allowing the construction of full-length infectious clones. This assembly strategy was rapidly deployed for the study of human pathogenic betacoronaviruses, including SARS-CoV, MERS-CoV and SARS-CoV-2 [131,132,133].

The use of reverse genetic systems has allowed targeted genetic manipulation of viral genes and creation of homogeneous viral stocks for running in vitro and in vivo assays. Based on their genomic organization, reporter strains of both SARS-CoV and MERS-CoV have been created by replacing the ORFs of accessory proteins with luciferase and fluorescent proteins as reporter genes [134,135,136,137]. These reporter strains, as well as mouse-adapted SARS-CoV and MERS-CoV clones, have been used to assess the role of individual mutations in host adaptation [136,137,138,139]. Similar studies based on reverse genetics have been critical in characterizing the function of several Nsps in replication and transcription, as well as modulation of host processes such as inflammatory responses during infection [19].

Reverse genetic systems are also useful to understand how the viruses evolve during outbreaks and epidemics. For instance, S protein mutations from zoonotic, early, middle and late epidemic strains of the SARS-CoV outbreak have been introduced into the S protein of the epidemic strain of SARS-CoV (Urbani) to evaluate the effect of those mutations on viral entry into human cells and viral pathogenesis in rodent and primate models [140,141,142]. In addition, reverse genetic systems have proven useful in analyzing the emergence and pathogenic potential of bat SARS-related and MERS-related coronaviruses [143,144,145,146]. Recombinant versions of bat betacoronaviruses can be used to evaluate the efficiency of the S protein-mediated viral entry and replication and to characterize genetic changes required for efficient infection of human cells.

### 5.2. Diagnostics

Methods based on polymerase chain reaction (PCR) are the most frequently used for detecting highly pathogenic human betacoronaviruses. These methods have several advantages including their high sensitivity and specificity, their feasibility in settings where virus isolation is not possible due to safety concerns and their ability to detect virus presence early after infection, even before the onset of symptoms [147,148]. After the 2002–2003 SARS-CoV outbreak, random-amplification deep-sequencing approaches have played a crucial role in discovery and characterization of genomic differences among SARS-related coronaviruses and identification of the emerging MERS-CoV and SARS-CoV-2 [73,149,150]. These studies allowed the rapid development of genus- and species-specific real-time PCR assays based on the genomes of these viruses. Available PCR tests for human pathogenic betacoronaviruses employ either a single or multiple primer sets targeting specific regions of the ORF1ab, E and N genes [147,148,151,152,153,154,155]. Although most of the assays developed using the aforementioned genes show no cross-reactivity with related species, and hence high specificity, assays targeting the N gene displayed higher sensitivity, probably because this transcript is very abundant during replication of betacoronaviruses [148,151,153,156,157]. In fact, even if relative abundance of subgenomic mRNAs is believed to be kept well controlled during the replicative cycle, an increasing gradient of expression has been reported from 5′ to 3′, with the N gene exhibiting the highest expression levels in cells infected with MHV [158].

Regardless of their efficiency, PCR-based assays have several drawbacks for their massive use during outbreaks and epidemics, including requiring specialized and costly equipment and reagents, as well as having turnaround times ranging from 2 to 4 days due to the time required for sample transportation to centralized testing facilities, preparation and performance of the actual PCR test. In recent years, a rapid molecular test has been developed to detect the N gene of MERS-CoV based on a combination of reverse transcription loop-mediated isothermal amplification and a vertical flow visualization strip (RT-LAMP-VF) [156]. This test exhibits no cross-reactivity with SARS-related coronaviruses and has a turnaround time of approximately 35 minutes. A new assay called DNA Endonuclease-Targeted CRISPR Trans Reporter (DETECTR) has been developed for SARS-CoV-2 detection, which can be performed in less than 40 minutes [157]. This assay performs simultaneous RT-LAMP for RNA samples followed by Cas12 detection using guide RNA sequences targeting species-specific regions of the E and N genes of SARS-CoV-2.

Immunoassays based on antigen-antibody recognition are an alternative for the establishment of point-of-care tests that deliver fast results at a low-cost, and are fundamental for providing diagnostic evidence and for better understanding of the epidemiology of emerging betacoronaviruses, including the burden of asymptomatic infections and exposure. Knowledge of the genomic sequences of infecting coronaviruses has been critical for the development and validation of immunoassays that either use monoclonal antibodies (mAbs) to detect viral antigens in clinical samples or cloned viral antigens to detect patient antibodies directed against the virus [147,159,160,161,162]. Development of antigen tests requires the expression of recombinant viral proteins or fragments of them that contain potential epitopes predicted by sequence homology to previously described immunogenic motifs [163,164]. These recombinant antigens are subsequently used for the production of specific mAbs, followed by experimental validation of their affinity for viral antigens and characterization of their specific epitopes [165].

Prototypes of direct antigen tests have been developed previously for SARS-CoV and MERS-CoV, but have not received regulatory approval, whereas SARS-CoV-2 antigen tests are currently under development [147,159,161,166]. Most of these antigen-based assays have targeted the N protein, since it is probably the most convenient target for virus detection in patients due to its high abundance. Serological assays that rely on recombinant proteins as antigens have been developed for detection of SARS-CoV, MERS-CoV and SARS-CoV-2, mainly using the N and S proteins as the two major immunogenic proteins of these viruses [160,162,167,168]. Although serological assays have limited utility for diagnostic purposes due to the variable time span for antibodies to be detectable after initial infection, these assays may be especially useful for unveiling the real epidemiological impact of pandemics such as the 2019–2020 COVID-19 pandemic, given the increasing evidence of highly abundant asymptomatic carriers [169,170,171].

### 5.3. Drug Design

When SARS-CoV suddenly emerged in late 2002, the initial approach to drug discovery was to test existing broad-spectrum antiviral drugs as potential anti-betacoronavirus candidates [172]. In addition to drug repurposing, a more rational approach widely used in the aftermath of SARS-CoV and MERS-CoV was structure-based drug design (reviewed by Hilgenfeld and Peiris [173]). Not surprisingly, similar strategies have also been explored since the beginning of the 2019–2020 COVID-19 pandemic [174]. Accelerated discovery of new SARS-related betacoronaviruses and characterization of their genomes have allowed the incorporation of genomic information into drug discovery pipelines. Reverse genetic systems have made possible biological assays for characterizing the function of viral proteins, the first important step for identification of potential virus-specific drug targets [131,132,133]. Both genomic and functional knowledge have allowed the development of small interfering RNA (siRNA) molecules targeting specific viral proteins. This strategy has been used to design siRNA inhibitors targeting the ORF1b and S genes of SARS-CoV [175] and has also been suggested as a valid strategy against SARS-CoV-2 [176].

Genomic knowledge also serves as the basis for other ‘Omics’ such as transcriptomics, proteomics and interactomics, which have also been crucial for accelerating drug discovery against SARS-CoV-2. Based on the high sequence similarity between SARS-CoV-2 and other human betacoronaviruses, especially SARS-CoV, network proximity analysis of drug targets and virus-host interactions in the human interactome has been already used as a tool for accelerating drug repurposing [177]. In this type of analysis, proteins functionally associated with viral infection are localized in the corresponding subnetwork within the human protein-protein interaction network, and those proteins that serve as drug targets for specific diseases are selected as potential targets for antiviral drugs. These analyses are followed by bioinformatic validation of drug-induced gene signatures and human betacoronavirus-induced transcriptomics in human cell lines to inspect the postulated mechanisms of action in a specific human betacoronavirus.

A high-resolution map of the SARS-CoV-2 transcriptome and epitranscriptome has been recently elucidated [178]. Data from this study revealed an overly complex transcriptome, characterized by a large number of transcripts encoding unknown ORFs produced by fusion, deletion and/or frameshift events. Furthermore, direct RNA sequencing suggested 41 potential RNA modification sites on the viral transcripts, the majority of them containing the AAGAA motif. Functional characterization of these newly discovered ORFs and RNA modification sites may unveil key roles in viral pathogenesis, defining new potential targets for antiviral therapy. In a collaborative effort, several research groups were able to clone, tag and express 26 of the 29 viral proteins found in human cells [179]. More than 300 high-confidence SARS-CoV-2-human protein-protein interactions were further identified using affinity-purification mass spectrometry (AP-MS). Applying a combination of systematic chemoinformatic drug search and pathway centric analysis to the whole set of interactions, 66 druggable human proteins were identified that are targeted by 69 existing approved drugs and compounds in clinical and/or pre-clinical trials.

### 5.4. Vaccine Candidates

In the aftermath of previous SARS and MERS outbreaks, several laboratories around the globe pursued the development of vaccines using the traditional strategy of inactivating whole viral particles (reviewed by Roper and Rehm [180] and Zumla et al. [172]). Increasing availability of genomic information regarding SARS-CoV, MERS-CoV and other related betacoronavirus have allowed the development of other types of vaccine formulations such as live-attenuated vaccines, recombinant vector vaccines and DNA vaccines. Reverse genetic systems have been used to develop and characterize live-attenuated vaccine platforms in both SARS-CoV and MERS-CoV, based on substitutions of key residues of the Nsp16 active site [181,182], deletion of the E gene [183,184,185,186] or inactivation of the exonuclease activity of Nsp14 [187]. Although showing promising results, these live-attenuated vaccines also raised safety concerns, due to the possibility of recombination and reversal of mutations that could restore the functionality of the inactivated proteins.

As an alternative to live-attenuated vaccines, recombinant vector vaccine candidates have been developed for SARS and MERS using either adenovirus [188,189,190,191,192,193,194], parainfluenza [195], vesicular stomatitis virus [196], attenuated measles virus [197], baculovirus [198], vaccinia modified virus Ankara [199,200,201] and attenuated *Salmonella* [202] as vectors for expression of S, E, M and N proteins. These recombinant vectors express the foreign target protein in the cytoplasm of the host cell, thus inducing both cellular and humoral immune responses. Following a similar principle, DNA and RNA vaccines can induce both B- and T-cell mediated immunity without the use of any viral particle, by simply introducing into the host cell plasmids encoding proteins of the pathogen that are then endogenously produced. Several DNA vaccine formulations against SARS-CoV and MERS-CoV, mainly based on the S, M and N proteins, showed promising results in the pre-clinical phase [203,204,205,206,207,208].

Many SARS-CoV and MERS-CoV vaccine formulations evaluated in animal models protected animals from challenge with the virus, but failed to induce protective immunity in aged groups and exacerbated SARS symptoms in younger groups subsequently challenged with the virus [209,210,211,212,213]. These findings stress the importance of developing subunit vaccines, as these will offer targeted immunogenicity with improved safety. For this purpose, bioinformatic tools can be used to predict potential epitopes in proteins encoded in the genomes, based on their sequence similarity to previously described immunogenic motifs or through structural methods such as molecular docking simulations. A recent study used predictive bioinformatic tools to identify potential B- and T-cell epitopes for SARS-CoV-2 in regions of its genome with high sequence similarity to SARS-CoV [164]. Epitopes derived from the S protein of human betacoronaviruses seem to be the most promising for the development of strong subunit vaccines, as it has been shown that the SARS-CoV S protein can induce serum-neutralizing antibodies [195,203] and generate CD4^+^ and CD8^+^ T-cell responses [214].

Thanks to the availability of SARS-CoV-2 genome sequences and the previous experience with SARS-CoV and MERS-CoV, numerous vaccine projects using diverse technologies are currently in progress, with some already entering into clinical trials (reviewed by Amanat and Krammer [215]). Evidence that neutralizing antibodies against SARS-CoV cross-react with SARS-CoV-2 suggests that SARS-CoV vaccines might cross-protect against SARS-CoV-2. Unfortunately, the few SARS-CoV vaccines that made it to phase I clinical trials were not further funded due to the control of the disease [215]. Noteworthy is that several of these abandoned projects for SARS-CoV vaccines have been reactivated and rapidly adapted to SARS-CoV-2. The relatively high sequence divergence between SARS-CoV-2 and MERS-CoV makes it unlikely that vaccines targeting MERS-CoV can induce strong cross-neutralizing antibodies against SARS-CoV-2. However, existing platforms for the development of MERS-CoV vaccines were also rapidly adapted for the production of SARS-CoV-2 vaccines, as is the case of the RNA-based vaccine developed by Moderna Therapeutics and the Vaccine Research Center at the National Institutes of Health (NIH), which encodes a segment of the S gene encapsulated in lipid-based nanoparticles (ClinicalTrials.gov: NCT04283461). The high genomic diversity observed in bat betacoronaviruses suggests that development of a pan-betacoronavirus vaccine will be unlikely; however, available technologies for vaccine production and rapid acquisition of genomic information can pave the way for the development of modular vaccine platforms that are rapidly adjustable to new antigens in potentially emerging epidemics [128].

## 6. Concluding Remarks

Since the 2002–2003 SARS outbreak, genomic information has been crucial to tackle epidemics caused by betacoronaviruses. During the 2019–2020 COVID-19 pandemic, quick availability of genomic data has allowed a very rapid, detailed and accurate follow-up of disease progression worldwide and has tremendously supported the development of diagnostic systems, drug candidates and vaccines. Full viral genome analysis has swiftly changed the way scientists deal with epidemic viruses in two main ways: first, the speed that allows the description and classification of the responsible pathogen in a record timeframe, and second, the ability to generate massive amounts of viral genome data, contributing to establishing sound hypotheses on evolution and transmission. It is remarkable that genome analyses at such a scale are now increasingly feasible, without having to culture the viruses, many of which are classified as Biosafety Level 3 agents. However, it is important to stress that genomic information must be used carefully when drawing conclusions related to human and animal health. Sampling bias, selection of inadequate bioinformatic tools and misinterpretation of results can all lead to unreliable conclusions. Furthermore, the quality of genomic sequences used in comparative analyses is also crucial to establish sound conclusions. Currently, there are several platforms used for genome sequencing, each of them with their own patterns of systematic sequencing errors. Data curation and normalization is extremely important before conducting further analyses, particularly when comparing data from different sequencing platforms. If analyzed properly, genomic data will indisputably serve as strong basis for addressing future outbreaks caused by highly pathogenic emerging viruses such as SARS-CoV-2.

## Supplementary Materials

The following are available online at https://www.mdpi.com/1422-0067/21/12/4546/s1, Table S1: Additional information on selected betacoronavirus genomes.
