# Supplementary material for: Betacoronavirus Genomes: How Genomic Information has been Used to Deal with Past Outbreaks and the COVID-19 Pandemic
Source: Int J Mol Sci. 2020 Jun 26;21(12):4546. doi: 10.3390/ijms21124546 (PMC7352669; doi:10.3390/ijms21124546)
Supplement: Supplementary file 1 [file ijms-21-04546-s001.zip › Table S1.pdf]

Table S1. Additional information on selected betacoronavirus genomes.

GenBank accession	Virus	Subgenus	Isolate/strain	Host	Date published	Authors
AY686863	SARS-CoV	*Sarbecovirus*	A022	*Paguma larvata*	06/2004	Xu et al.
DQ011855	Porcine hemagglutinating encephalomyelitis virus (PHEV)	*Embecovirus*	VW572	*Sus scrofa*	04/2005	Vijgen et al.
DQ022305	Bat SARS-like CoV HKU3	*Sarbecovirus*	HKU3-1	*Rhinolophus sinicus*	04/2005	Lau et al.
KF367457	Bat SARS-like CoV WIV1	*Sarbecovirus*	WIV1	*Rhinolophus sinicus*	07/2013	Ge et al.
KF906249	Dromedary CoV HKU23	*Embecovirus*	HKU23-265F	*Camelus dromedarius*	11/2013	Woo et al.
KF917527	MERS-CoV	*Merbecovirus*	MERS-CoV-Jeddah-Camel-1	*Camelus dromedarius*	12/2013	Azhar et al.
MF593268	*Neoromicia* bat CoV	*Merbecovirus*	Neoromicia/5038	*Neoromicia capensis*	07/2017	Geldenhuys et al.
MG772933	Bat SARS-like CoV SL-CoVZC45	*Sarbecovirus*	Bat-SL-CoVZC45	*Rhinolophus sinicus*	01/2018	Hu et al.
MG772934	Bat SARS-like CoV SL-CoVZXC21	*Sarbecovirus*	Bat- SL-CoVZXC21	*Rhinolophus sinicus*	01/2018	Hu et al.
MK907286	Hedgehog CoV HKU31	*Merbecovirus*	F6	*Erinaceus amurensis*	05/2019	Lau et al.
MN996532	Bat SARS-like CoV RaTG13	*Sarbecovirus*	RaTG13	*Rhinolophus affinis*	01/2020	Zhu et al.
MT040333	Pangolin CoV	*Sarbecovirus*	PCoV_GX-P4L	*Manis javanica*	02/2020	Cao et al.
MT365033	SARS-CoV-2	*Sarbecovirus*	SARS-CoV-2/ tiger/NY/040420/2020	*Panthera tigris jacksoni*	04/2020	Mitchell et al.
NC_003045	Bovine CoV	*Embecovirus*	BCoV-ENT	*Bos taurus*	08/2001	Chouljenko et al.
NC_004718	SARS-CoV	*Sarbecovirus*	Tor2	*Homo sapiens*	04/2003	BCCA Genome Sciences Centre, Canada (Consortium)
NC_006213	Human CoV OC43	*Embecovirus*	ATCC VR-759	*Homo sapiens*	03/2004	St-Jean et al.
NC_001846	Mouse hepatitis virus (MHV)	*Embecovirus*	MHV-A59	*Mus musculus*	10/1997	Weiss et al.
NC_006577	Human CoV HKU1	*Embecovirus*	HKU1	*Homo sapiens*	04/2004	Woo et al.
NC_009019	*Tylonycteris* bat CoV HKU4	*Merbecovirus*	HKU4-1 B04f	*Tylonycteris* sp.	10/2006	Woo et al.

NC_009020	*Pipistrellus* bat CoV HKU5	*Merbecovirus*	HKU5-1 LMH03f	*Pipistrellus* sp.	10/2006	Woo et al.
NC_009021	*Rousettus* bat CoV HKU9	*Nobecovirus*	HKU9-1 BF_005I	*Rousettus* sp.	10/2006	Woo et al.
NC_012936	Rat CoV Parker	*Embecovirus*	Parker	*Rattus norvegicus*	04/2009	Spiro et al.
NC_019843	MERS-CoV	*Merbecovirus*	EMC/2012	*Homo sapiens*	10/2012	van Boheemen et al.
NC_025217	Bat Hp-BetaCoV Zhejiang2013	*Hibecovirus*	Zhejiang2013	*Hipposideros pratti*	09/2013	Wu et al.
NC_026011	China *Rattus* CoV HKU24	*Embecovirus*	HKU24-R05005I	*Rattus norvegicus*	08/2014	Lau et al.
NC_030886	*Rousettus* bat CoV GCCDC1	*Nobecovirus*	GCCDC1 356	*Rousettus leschenaulti*	02/2006	Huang et al.
NC_045512	SARS-CoV-2	*Sarbecovirus*	Wuhan-Hu-1	*Homo sapiens*	01/2020	Wu et al.
